# Systematic comparison of mStayGold and mGold2 variants for live imaging in zebrafish

**DOI:** 10.17912/micropub.biology.001847

**Published:** 2025-11-25

**Authors:** Aline Tschanz, David Q. Matus, Ian Swinburne

**Affiliations:** 1 MCB, University of California, Berkeley, Berkeley, California, United States

## Abstract

Fluorescent proteins (FPs) are essential tools for live imaging, with brightness and photostability being key parameters when selecting an FP for experiments. Recently developed variants of monomeric StayGold (mSG) and mGold2 promise improvements in these properties, yet their performance can vary across model organisms and tissue contexts. To guide the selection of FPs for zebrafish imaging, we expressed membrane-targeted FP fusions in embryos and systematically compared their *in vivo* brightness and photostability against the widely used mNeonGreen (mNG). Among the mSG variants, mSG(A) displayed brightness comparable to mNG but with markedly increased photostability. In contrast, mSG(BJ) and mSG(E138D) showed reduced brightness, while retaining improved photostability. The mGold2 variants exhibited no improvement in photostability over mNG, but their increased brightness at 514 nm excitation allowed effective imaging at lower laser intensities, thereby extending usable imaging times. There were no significant differences detected between the two mGold2 variants. Overall, mSG(A) emerges as an optimal choice for long-term imaging, while mGold2 variants are advantageous when maximal brightness is required. These results provide practical benchmarks for FP selection in zebrafish embryonic imaging.

**Figure 1. Systematic comparison of monomeric StayGold (mSG) in living zebrafish embryos:**
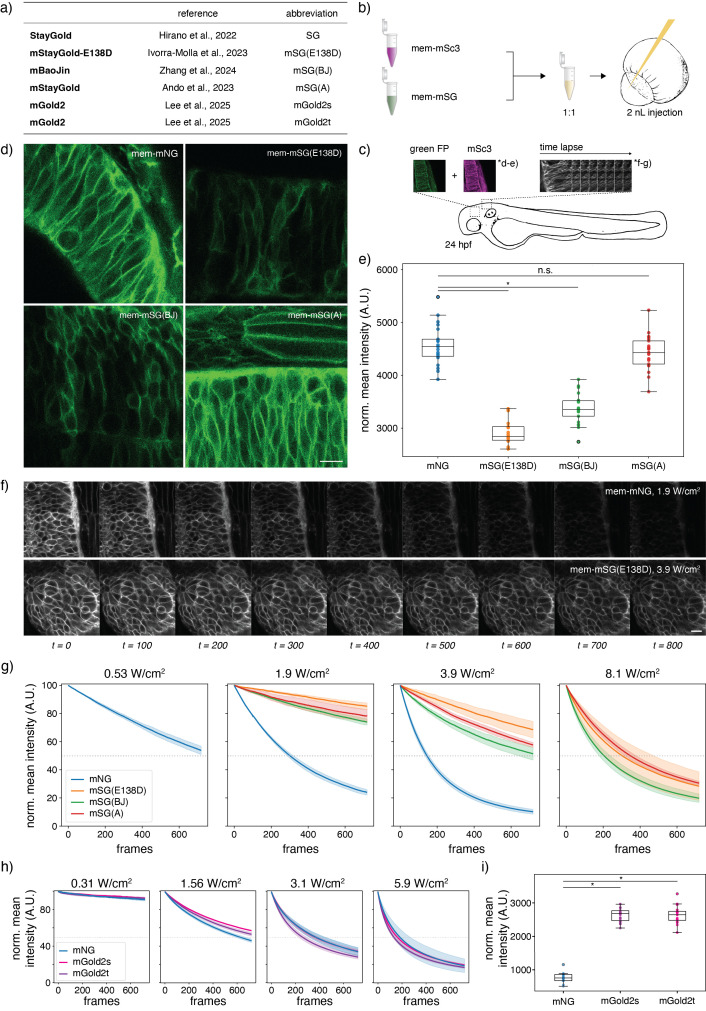
a) Three distinct monomeric variants of the original StayGold (SG) fluorescent protein have recently been published. b) Transient expression of monomeric SG (mSG) by mRNA injection. mSG is targeted to the plasma membrane (mem-mSG) by fusing it to two copies of the membrane associated palmitoylation-myristoylation motif of Lyn kinase. mRNA of mem-mSG and equally modified mem-mScarlet3 are mixed at 1:1 ratio and injected into the zebrafish embryo at the one cell stage. c) Embryos were imaged at 24 hpf. Dual-color snapshots of mem-mSG and mem-mSc3 were acquired to measure FP brightness and time lapse imaging was performed to assess bleaching behavior of FPs. d) Example images of mNeonGreen and three mSG variants, normalized to the average signal intensity of the respective mSc3 frame to account for injection variability. e) Normalized mean intensity calculated for mNG and each mSG variant. Error bars are standard deviation. Kruskal–Wallis test, with Dunn’s multiple comparisons test. *P < 0.001; n.s. not significant. (N = 21 embryos; n = 3 snapshots per embryo). f) Example images from time lapse acquisition, showing mNG and mSG(E138D) imaged at different laser powers for similar image quality. 9 of 800 frames shown. g) Quantification of time lapse imaging for mNG and mSG. Each condition was acquired in triplicates. Average intensity (solid line) and standard deviation (shaded) across these replicates are plotted per FP. Dotted grey line represents half the initial signal intensity. Signal intensities were normalized to 100 at t0 for comparability, and the first and last 5% of frames neglected from the analysis. Measured at 1.9 W/cm ^2^ . h) Normalized mean intensity calculated for mNG and each mGold2 variant. Error bars are standard deviation. Kruskal–Wallis test, with Dunn’s multiple comparisons test. *P < 0.001; n.s. not significant. (N = 20 embryos; n = 3 snapshots per embryo). i) Quantification of time lapse imaging for mNG and mGold2, same method as in g, measured at 0.31 W/cm ^2^ . Scale bars: 10 µm.

## Description

Fluorescent proteins (FP’s) isolated from nature and their derivatives have become indispensable tools for visualizing dynamic cellular processes in living cells and organisms. Since the discovery of Green Fluorescent Protein (GFP) in the 1960s, continuous advancements in protein engineering have led to significant improvements in fusion compatibility, brightness and photostability, enabling increasingly sensitive and prolonged imaging experiments. Recently, novel FP variants have been published, promising increased photostability and brightness. These include three monomeric versions of the green fluorescent protein StayGold (SG), originally isolated from the cnidarian hydrozoan, *Cytaeis uchidae* (Ando et al. 2024; Zhang et al. 2024; Ivorra-Molla et al. 2024) (Figure 1a), and two monomeric versions of the yellow fluorescent protein mGold2, a mVenus-derived FP(Lee et al. 2025). The monomeric SG (mSG) variants have been individually validated in vitro, yeast and mammalian cells, fixed mouse brain tissue (Ando et al. 2024; Zhang et al. 2024; Ivorra-Molla et al. 2024), and *C. elegans* (Ko and Mizumoto 2025), and both mGold2 variants were tested in yeast and mammalian cells (Lee et al. 2025). While these results promise similar advances in brightness and photostability, the variants vary in sequence and photophysical properties. These small discrepancies can lead to significant performance differences in complex tissue environments, particularly as organisms evolved distinct optimal incubation temperatures, such as developing zebrafish embryos.

To support informed selection of suitable fluorescent proteins (FPs) for live imaging in zebrafish, we systematically compared the performance of published mSG and mGold2 variants across distinct embryonic tissues. We benchmarked their performance against the widely used mNeonGreen (mNG), focusing on in vivo brightness and photostability.

For this purpose, we transiently expressed fusion proteins, where each FP is fused to two copies of the membrane associated palmitoylation-myristoylation motif of Lyn kinase (2xLynk, iCodon optimized). We injected mRNA encoding each FP fusion at the one-cell stage, together with red membrane-targeted mScarlet3 (mSc3) to control for variation in injection volume (Figure 1b). We then assessed relative brightness and photostability in 24 hours-post fertilization (hpf) zebrafish embryos by acquiring snapshots and time lapses across different tissues (Figure 1c).

To quantify their relative brightness, we acquired images across 21 embryos per FP. For each embryo, images were acquired in triplicates, and normalized by the average signal intensity of their respective mSc3 channel (Figure 1d). Comparing the average signal intensity per embryo reveals similar brightness between the mSG(A) and mNG. mSG(BJ) and mSG(E138D) data show significantly reduced brightness relative to mSG(A) and mNG (Figure 1e).

Taking 800 consecutive frames at different laser powers, we next assessed the bleaching behaviour of the mSG variants and mNG (Figure 1f). As mNG is approximately twice as bright as mSG(E138D), comparable image qualities require approximately twice the laser power (see first frame in Figure 1f). However, even when increasing the laser power from 1.9 to 3.9 W/cm ^2^ , mSG is still markedly more photostable than mNG barely approaching half the initial intensity by the end of the image acquisition (Figure 1g). To achieve a similar stability when imaging with mNG, laser power had to be reduced to as low as 0.53 W/cm ^2^ , where image quality is insufficient. Bleaching of mSG was only realized when increasing the laser power to 8.1 W/cm ^2^ .

We repeated the same experiments to test the performance of mGold2 variants in zebrafish embryos (Figure 1h-i). Our results showed no improvement in photostability of either mGold2 variant, both of which followed the same bleaching trend as mNG (Figure 1h). However, the significant increase in brightness of both mGold2 variants (partially due to optimal excitation with our 514 nm laser) enables users to image at lower laser intensities for similar image quality, thus effectively prolonging imaging duration with these FPs (Figure 1i).

Taken together, all mSG variants show improved photostability, with mSG(A) being as bright as mNG when optimized for zebrafish expression. This makes mSG(A) an ideal candidate for long-term imaging. For applications where exceptional brightness might be more relevant than intrinsic photostability, both mGold2 variants perform remarkably well. For our further work we have selected both mSG(A) and either mGold2 as our preferred FPs in the green-yellow spectrum.

## Methods

Zebrafish strain

Zebrafish were maintained at 28.5 °C in the UC Berkeley zebrafish facility, under the supervision of ACUC protocols (AUP-2020-10-13737, last approval date 12/18/2024). Embryos of 2-year-old adult Casper (mitfa^w2/w2; mpv17^a9/a9) (White et al. 2008) mutant in-crosses were used in this study.

Plasmids

Nucleotide sequences codon optimized for expression in zebrafish, corresponding to mSG and mGold2 variants, and mNG, were designed in silico using iCodon (Diez et al. 2022) and synthesized at Twist Bioscience. Gene blocks were cloned into an expression vector (pTwist Amp) using Gibson assembly (Gibson et al. 2009). Briefly, plasmid pDQM048 (2xLynk:mSG(A), Addgene plasmid # 247634) was generated containing a 126bp fragment corresponding to two copies of a Lyn Kinase membrane localization sequence (Megason and Fraser 2003) fused to mSG(A) separated by a BamHI restriction site encoding a Glycine-Serine linker. The remaining plasmids were generated by BamHI/NheI restriction digest of pDQM048 and insertion of iCodon optimized sequences of the other FP gene blocks by Gibson assembly, corresponding to plasmids pAT002 (2xLynk:mNeonGreen, Addgene plasmid # 248842), pDQM045 (2xLynk:mScarlet3, Addgene plasmid # 247633), pDQM049 (2xLynk:mSG(E138D), Addgene plasmid # 247635), pAT005 (2xLynk:mBaoJin, Addgene plasmid # 247636), pDQM138 (2xLynk:mGold2s, Addgene plasmid # 247637), and pDQM139 (2xLynk:mGold2t, Addgene plasmid # 247638). Plasmids were sequence verified using whole plasmid sequencing (Plasmidosaurus). Plasmids are available at Addgene.

mRNA

*In-vitro* transcription of plasmids was performed using standard protocols under RNase-free conditions. Plasmids were linearized using MfeI restriction enzyme (NEB), followed by a PCR clean-up using Monarch® PCR & DNA Cleanup Kit (New England Biolabs) according to the manufacturer’s recommendations.

mRNA was synthesized using SP6 mMessage mMachine kit (Thermo Fisher Scientific) following the manufacturer’s protocol. Following synthesis, mRNA clean-up was performed using RNeasy Mini Kit (50) (Qiagen) according to the manufacturer’s protocol.

After clean-up RNA concentration was measured using a NanoDrop spectrophotometer, adjusted and aliquoted at 200 ng/µL, and stored at -80C.

mRNA injection

For injection solution, mem:mSc3 mRNA was combined with mRNA of either of the green FP (mem:mSG(B); mem:mSG(BJ); mem:mSG(J); mem:mNG) and diluted in RNase-free water and injection buffer (0.1 M KCl; 0.1% phenol red salt; 0.1 mM EDTA; 1 mM Tris pH 7.) to a final concentration of 20 ng/µL per construct.Embryos were injected at the one-cell stage directly into the embryonic cell at a volume of 2 nL and injection speed of 10 nL/s using the Nanoject III® Programmable Nanoliter Injector (Drummond Scientific). Non-viable injected embryos were screened and discarded approximately 3 hours after injection.

Imaging

Imaging was performed on an upright LSM 980, 3 channel, 1GaAsP detector, 2 PMT detectors (Zeiss)). 24 hpf embryos were immobilized using 3X Tricaine buffer (75 mg tricaine powder; 0.75 mL 0.3M Tris, pH 8.0; 0.075 mL NaOH; 73.35 mL 1X Danieau buffer (“Danieau’s Solution (30×)” 2011) and mounted in a submerged agarose canyon (400 μm wide) (Swinburne et al. 2015). No. 0 22x22 mm coverslips were placed on the embryos for stabilization. A 20X/1.0 NA water immersion objective was used for imaging.

Brightness measurements

For each green FP condition, 21 embryos were imaged in triplicates. For each snapshot, the frame size was 512 px x 512 px, at a pixel size of 0.28 µm and 16 bits per pixel. Frame time was 314.57 ms at a pixel time of 2.05 µs. For each snapshot of a green FP at 488 nm excitation, a reference image of mSc3 was acquired at 561 nm excitation. 488 nm laser was run at 3%, corresponding to a laser intensity of 100 µW measured after the objective (0.5 W/cm ^2^ for illumination area of 20’552 µm ^2^ ). 561 nm laser was run at 0.5%, corresponding to 23 µW after the objective (0.1 W/cm ^2^ ).

For mGold2, 20 embryos were imaged in triplicates. For each snapshot, the frame size was 512 px x 512 px, at a pixel size of 0.28 µm and 16 bits per pixel. Frame time was 314.57 ms at a pixel time of 2.05 µs. For each snapshot of a yellow FP at 514 nm excitation, a reference image of mSc3 was acquired at 561 nm excitation. 514 nm laser was run at 0.5%, corresponding to a laser intensity of 16 µW measured after the objective (0.08 W/cm ^2^ for illumination area of 20’552 µm ^2^ ). 561 nm laser was run at 0.5%, corresponding to 23 µW after the objective (0.1 W/cm ^2^ ).

Time lapse imaging

800 consecutive frames were acquired at 4 different laser power conditions. Each condition was performed in triplicates (covering 2-3 embryos per condition). The frame size was 256 px x 256 px, at a pixel size of 0.28 µm and 16 bits per pixel (illumination area of 5138 µm ^2^ ). Frame time was 314.57 ms at a pixel time of 2.05 µs and imaging was performed in confocal detection mode at a 0.5x sampling rate. The 488 nm laser was run at 1% (28 µW, 0.53 W/cm ^2^ ), 3% (100 µW, 1.9 W/cm ^2^ ), 6% (202 µW, 3.9 W/cm ^2^ ), and 12% (414 µW, 8.1 W/cm ^2^ ) respectively. The 514 nm laser was run at 0.5% (16 µW, 0.31 W/cm ^2^ ), 2.5% (80 µW, 1.56 W/cm ^2^ ), 5% (160 µW, 3.1 W/cm ^2^ ), and 10% (305 µW, 5.9 W/cm ^2^ ).

Analysis

Analysis code is available on GitHub (https://github.com/alinetschanz/mStayGold_analysis). Average background intensity was estimated by acquiring an image without laser activity and away from the sample in both 488 nm and 561 nm channels. The average frame intensity was then subtracted from the imaging data. Average intensity per frame was calculated after background subtraction. Normalization was performed to account for mRNA injection volume variability. A normalized image (norm) of a red FP image (img) was generated using the signal of the corresponding mNG z-slice (ref): norm= (img/ref )*img . For each z-stack the mean intensity across the entire normalized z-stack was calculated. Average intensity per frame was calculated after background subtraction. Signal intensities were normalized to 100 at t0 for comparability, and the first and last 5% of frames neglected from the analysis. Mean signal intensities were calculated across three replicates.
